# Characteristics of lactate metabolism phenotype in hepatocellular carcinoma

**DOI:** 10.1038/s41598-023-47065-0

**Published:** 2023-11-11

**Authors:** Jiacheng Zhang, Keshuai Dong, Xin Zhang, Chunlei Li, Jia Yu, Weixing Wang

**Affiliations:** 1 Department of Hepatobiliary Surgery, Renmin Hospital of Wuhan University, Wuhan, 430060 Hubei People’s Republic of China; 2 Central Laboratory, Renmin Hospital of Wuhan University, Wuhan, 430060 Hubei People’s Republic of China; 3 Department of General Surgery, Renmin Hospital of Wuhan University, Wuhan, 430060 Hubei People’s Republic of China

**Keywords:** Prognostic markers, Tumour biomarkers, Liver cancer

## Abstract

Hepatocellular carcinoma (HCC) is a highly heterogeneous cancer, and more effective prognostic markers are needed. Lactic acid has been proved to be an important metabolite involved in cancer development, metastasis, and the tumor microenvironment, affecting the prognosis of patients. The role of lactic acid metabolism regulators (LAMRs) in HCC is still unclear. In this study, we analyzed the status of LAMRs, a gene list containing lactate from Molecular Signatures database, in HCC and consensus clustering was performed based on these LAMRs. Cluster B showed higher infiltrations of immune cells, higher TME scores, and a poorer prognosis. We further constructed a risk score based on DEGs using LASSO and COX regression analysis between two clusters, which could effectively predict the prognosis of TCGA-LIHC patients. The GSE14520 cohort confirmed the result. We also examined the correlation of risk scores with clinical characteristics, genetic mutations, drug sensitivity, immune checkpoint inhibitors(ICIs), and immunotherapy. In conclusion, our findings will facilitate a further understanding of the role of partial lactate metabolism related genes in HCC and suggest a new risk score to predict prognosis.

## Introduction

Primary liver cancer is one of the most common malignant tumors in the world. It is the third leading cause of cancer death worldwide, and among them, hepatocellular carcinoma (HCC) accounts for more than 80% HCC^1,2^. The major risk factors for HCC include chronic infections with the hepatitis B virus (HBV) and hepatitis C virus (HCV). In addition, excessive alcohol consumption, long-term smoking, obesity, and genetics are also important factors that can cause HCC. The late diagnosis, high metastasis rate, and high recurrence rate ultimately lead to a high mortality rate of HCC^3^. Many clinical indicators help predict patient prognosis, including AFP, TNM staging, and vascular invasion^4^. However, considering the great heterogeneity of HCC, novel prognostic models are needed.

Metabolic changes in tumor cells are beneficial to cell proliferation and have profound effects on tumor immunity through the release of metabolites, especially lactate. Otto Warburg^5^ first described that tumor cells consumed substantial glucose and secreted lactate even under aerobic conditions in the 1920s, which is now known as the Warburg effect, and also is a hallmark characteristic of cancer^6^. Numerous studies have provided evidence that lactate plays an integral role in tumor progression, providing a survival advantage to tumor cells through the upregulation of oncogenes, induction of angiogenesis, local infiltration of tumor cells, and distant metastasis^7,8^. Metastasis, tumor recurrence, and low survival in cervical cancer are closely associated with high lactate levels in the tumor microenvironment^9^. Lactic acid and the resulting acidic tumor microenvironment (TME) also promote cancer cell immune evasion^7,10^. There is a strong link between lactic acid and immunity. Lactic acid promotes the expression of regulatory T cells' programmed cell death protein 1 (PD-1) in the microenvironment of high glycolytic tumors, and found that high glycolytic tumors consume glucose, release excessive LA, increase the expression of PD-1, and inhibit the activity of Treg cells, resulting in the ineffectiveness of partial PD-1 blocking therapy^11^. At present, machine learning and bioinformatics analysis have achieved remarkable results in many fields^12,13^, and the role of lactate-related genes in liver cancer analysis by bioinformatics remains to be further studied.

In the present study, we first discussed the expression, mutation, and internal relationship of 25 lactic acid metabolism regulators (LAMRs), a gene list containing lactate from Gene Ontology and HP. Then we identified two clusters related to lactate metabolism based on 25 LAMRs mRNA expression using consensus clustering analysis. And we also described the relationship between two clusters and clinical features and immune infiltration. Patients were then divided into two gene subtypes based on differentially expressed genes (DEGs) identified by two LAMR clusters. Furthermore, we developed a risk score to predict OS and further explored the relationship between the risk score and mutations, drug sensitivity, immune checkpoints, and immunotherapy.

## Methods

### Collection of LAMRs

Figure S1 showed the workflow of the present study. The 25 LAMRs were retrieved from the Molecular Signatures database, including GOBP_LACTATE_METABOLIC_PROCESS, GOMF_LACTATE_TRANSMEMBRANE_TRANSPORTER_ACTIVITY, and GOBP_LACTATE_TRANSMEMBRANE_TRANSPORT. In total, 25 Lactic acid metabolism regulators including ACTN3, HAGH, HIF1A, LDHA, LDHAL6A, LDHAL6B, LDHB, LDHC, LDHD, MIR210, PARK7, PER2, PFKFB2, PNKD, SLC16A1, SLC16A3, SLC16A7, SLC16A8, SLC25A12, SLC5A12, SLC5A8, TIGAR, TP53, MYC, EMB were included in this study. The basic information on the included Lactic acid metabolism regulators was shown in Table S1.

### Data processing

RNA transcriptome sequencing data and clinical data of patients were obtained from the TCGA-LIHC (https://portal.gdc.cancer.gov/) and GSE14520 (http://www.ncbi.nlm.nih.gov/geo). Somatic mutation data of HCC was also obtained from the TCGA. The FPKM values were normalized with the transcripts per million (TPM) method and then converted (log2 + 1) in TCGA. In total, 365 LIHC and 50 normal liver tissues were included in this study. The single-nucleotide variants (SNV) data of LIHC which containing 158 samples were also obtained from TCGA database. Copy Number Variation (CNV) data were downloaded from UCSC website(http://genome.ucsc.edu/). GISTIC was applied to calculate the CNV variation type (gain or loss) and frequency of LAMRs in the TCGA-LIHC cohort. We further studied the protein expression levels of LAMRs by IHC retrieving them from The Human Protein Atlas database (https://www.proteinatlas.org/). 6 to 12 LIHC samples from the 25 LAMRs were included and analyzed.

### mRNA and protein expression level of LAMRs

The difference in mRNA expression of LAMRs between HCC and normal liver samples in TCGA-LIHC was realized by the “limma” package. The protein levels of LAMRs expression in HCC samples were determined from the Human Protein Atlas (HPA)^14^, a website that contained immunohistochemical expression data for approximately 20 most common types of tumors.

### Functional annotation analysis and correlation of LAMRs

The LAMRs and subsequent genotyping of differential genes were then analyzed by Functional Annotation. To investigate the biological functions of these regulators, the “clusterProfiler” R package was used to carry out Gene Ontology (GO) functional annotations and Kyoto Encyclopedia of Genes and Genomes (KEGG) analysis (https://www.kegg.jp/kegg/kegg1.html). Also, to explore the interactions among the LAMRs, we calculated the Pearson correlation coefficient among LAMRs by using their expression in TCGA-LIHC data. The r package “corrplot” was used for visualization. We uploaded the list of 25 LAMRs to the STRING portal (https://string-db.org/) to build a protein–protein interaction (PPI) network, which could show the correlation between LAMRs^15^. Visualization of molecular interaction networks for PPI analysis was analyzed using the opensource software platform Cytoscape software (Cytoscape3.8.2)^16^. The degree of genes was further calculated by a tool in Cytoscape software named network analyzer to quantify the relationships among LAMRs.

### Consensus clustering analysis

Based on the expression of LAMRs in 365 HCC tissues, the “ConsensusClusterPlus” package was used to consensus cluster. To be specific, using agglomerative km clustering with a euclidean distances and resampling 80% of the samples for 1000 repetitions.The number of clusters ranged from 2 to 9. The consensus matrix and cumulative distribution function (CDF) were used to calculate the optimal number of clusters. The difference in overall survival (OS) between the two groups was assessed by the Kaplan–Meier method.

### Construction and validation of prognostic LAMR riskscore

The “limma” package was used to determine the DEGs between two clusters in TCGA-LIHC. |log_2_FC|≥ 1.5 and false discovery rate(FDR) < 0.05 were considered as the inclusion criterion. GO and KEGG enrichment analyses were performed for DEGs to analyze the biological behavior of these genes. Univariate Cox analysis was then used to determine whether DEGs were associated with prognosis (P < 0.05). Second, 365 LIHC samples were divided into two different subtype groups (lactic acid gene cluster A, and lactic acid gene cluster B) for deeper analysis. At the same time, DEGs with prognostic value were selected for further analysis. The least absolute shrinkage and selection operator (LASSO) Cox regression algorithm, to avoid overfitting, was used to perform regression analysis on the selected genes. The identification of candidate genes was subsequently achieved by utilising the optimal penalty parameter λ, employing the 1 − SE (standard error) criterion. The resulting genes were then subjected to multifactor regression analysis. Finally, based on the results of multivariate regression analysis^17^, a prognostic risk scoring system was calculated. The risk score was calculated using the following formula:

$$\mathrm{riskScore }= \sum_{i=1}^{n}Coef(i)*x(i)$$

where *Coef*(*i*) and *x*(*i*) were multivariate regression coefficients and the expression of the gene, respectively. Based on the median risk score, 365 HCC samples were divided into high- and low-risk groups. The risk score distribution, heatmap of genes included in the model, survival curve, and receiver operating characteristic (ROC) curve were analyzed. The relationship between risk-score and clinical features, such as age, gender, grade, T, and stage, was also evaluated. The efficacy of the risk score in predicting clinical outcomes was validated using GSE14520 as external data. In brief, the expression of genes included in the model in GSE14520 was selected, and the LAMR risk score for each sample was calculated according to the previous formula. According to the median score, they were divided into high and low risk groups for subsequent analysis. For TCGA-LIHC and GSE14520, the independent prognostic value of this feature was assessed by univariate Cox regression analysis and multivariate Cox regression analysis using the "survival" R package. The variables for analysis included age, gender, grade, TNM stage, and risk score.

### Tissue samples

Fifteen pairs of hepatocellular carcinoma and paraneoplastic tissues were obtained from patients undergoing surgery at the Department of Hepatobiliary Surgery, Renmin Hospital of Wuhan University. (Wuhan, China). The study was approved by the Ethics Committee of the Renmin Hospital of Wuhan University. The study has obtained the informed consent of all participants and/or their legal guardians and all experiments were performed in accordance with relevant guidelines and regulations.

### Quantitative real-time polymerase chain reaction PCR (RT-qPCR)

Total RNA was extracted from HCC and paraneoplastic tissues using TRIzol reagent (Servicebio, China). After RNA extraction, complementary DNA (cDNA) was synthesized using total RNA and a PrimeScript RT reagent kit (Takara). RT-qPCR was performed on a CFX-96 instrument (Roche LightCycler 480 II, Switzerland) using SYBR Green Master Mix (Servicebio, China). Primer sequences for qRT-PCR are listed in Supplementary Table S2. Gene expression was normalized to β-actin and calculated using the 2^−ΔΔCT^ method.

### Immunohistochemistry (IHC) analysis

IHC was conducted using antibodies against PHLDA2 from Proteintech Group Inc. (14661-1-AP, 1:1000, Chicago, USA). Briefly, sections were deparaffinized with xylene and ethanol and rehydrated before antigen retrieval by heating to just below the boiling temperature in Tris/EDTA buffer (pH 9.0) for 20 min in a microwave oven. Images were acquired using the 3DHIESTECH scanning system and software. The mean integrated option density (IOD) of PHLDA2-expressing in each sample was analyzed by Image-Pro Plus 6.0 software (Media Cybernetics Inc, Bethesda, USA).

### Western blotting analysis

Tissues were lysed in RIPA (Servicebio, G2002, China) buffer with proteinase inhibitor (Servicebio, G2008, China). The protein from each group was separated by 8–12% SDS-PAGE and transferred onto a cut PVDF membrane (Bio-Rad Laboratory). The membranes were blocked in a protein-free rapid blocking buffer (Epizyme, PS108, China). Then the membrane was incubated with specific antibodies overnight at 4℃, and probed with appropriate secondary antibodies. The signals were visualized using Bio-Rad ChemiDoc MP. Band intensities were quantified using ImageLab. The protein Gapdh was used as the loading control. The primary antibodies used for WB analysis included PHLDA2 (14661-1-AP, 1:1000) from Proteintech Group Inc. (Chicago, IL, USA) and GAPDH (GB15004, 1:1000, Servicebio, Wuhan, China).

### Establishment of a nomogram

Nomogram have been used to predict the prognosis of various cancers by incorporating the clinical characteristics and risk scores of the patients^18^. The nomogram is a predictive tool for many tumors prognosis and has advantages over traditional clinically relevant indicators^19^. It improves predictive power and can be used for patient identification and stratification^20^. In this study, Age, gender, grade, stage, and risk were used to construct the nomogram for evaluating OS in HCC. Validation of the ability of nomogram to predict prognosis using calibration and ROC curves.

### Mutation and drug-sensitivity analysis

The mutation annotation format (MAF) from the TCGA-LIHC was generated using the “maftools” R package to identify the difference in somatic mutations of HCC patients between high- and low-risk groups. To study the differences in the sensitivity of chemotherapeutic agents commonly used to treat HCC between the two groups, we employed the “pRRophetic” package to calculate the semi-inhibitory concentration (IC50) values.

### Expression of immune-related genes and immunotherapeutic responses

48 ICIs were retrieved from published literature and the expression of these ICIs was analyzed between high- and low-risk groups to evaluate the potential effects of risk score on the immunotherapy response. The correlation of the prognostic risk-score with two of the most widely used ICIs, PD-1 and cytotoxic T-lymphocyte-associated antigen 4 (CTLA4) was also determined. Data on immunotherapy for liver cancer are still not available, we used the IMvigor210 dataset derived from immunotherapy to further validate the response of this risk score to immunotherapy.

### Statistical analyses

R version 4.1.0 was used to perform statistical analyses and statistical significance was set at p < 0.05.

## Results

### The mRNA and protein expression and genetic alterations of LAMRs in HCC

**Figure 1:**
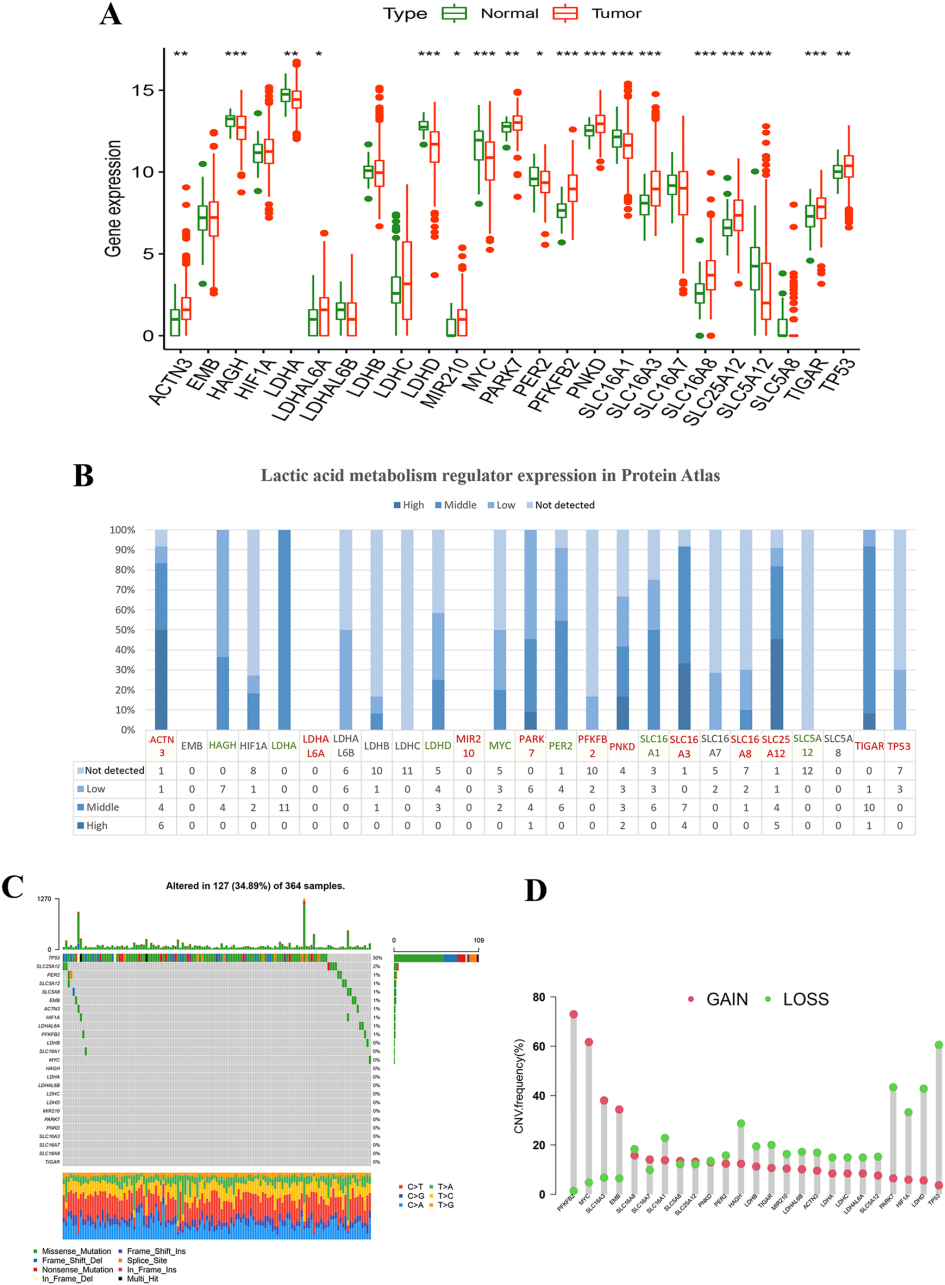
The mRNA and protein expression and genetic alterations of LAMRs in HCC. (**A**) mRNA expression of LAMRs, (**B**) protein expression of LAMRs, (**C**) mutation landscape map of LAMRs. (**D**) Frequencies of CNV gain, loss, and non-CNV among LAMRs.

Firstly, the mRNA expression of LAMRs between HCC and normal tissues was compared using the TCGA_LIHC cohort. As shown in the boxplot, 18 LAMRs were significantly different between the tumor and normal group, including ACTN3, LDHAL6A, MIR210, PARK7, PFKFB2, PNKD, SLC16A3, SLC16A8, SLC25A12, TIGAR, TP53 were upregulated, and HAGH, LDHA, LDHD, MYC, PER2, SLC16A1, SLC5A12 were downregulated significantly (Fig. 1A). The protein level of LAMRs was shown in the histogram (Fig. 1B). Red-labeled LAMRs represent up-regulated in the transcriptional level and green represents downregulated according to Fig. 1A. Consistent with mRNA levels, ACTN3, SLC16A3, SLC25A12, and TIGAR were shown high-expressed or middle expressed, HAGH, and SLC5A12 were shown low-expressed or not detected mostly. Part of protein expression was inconsistent with mRNA level. PFKFB and TP53 were in low expression and LDHA was in middle expression (Fig. 1B).

Next, the incidence of somatic mutations and copy number alterations were analyzed overall. Of the 364 LIHC samples, 127 (34.89%) had mutations in the LAMRs. Except for TP53, all other LAMRs showed a low mutation rate (Fig. 1C). We also found that the somatic copy number of these LAMRs was changed. Among them, the copy number variation (CNV) of PFKFB2, MYC, SLC16A3, and EMB generally increased, while the CNV of TP53, LDHD, HIF1A, PARK7, and HAGH decreased (Fig. 1D).

### Functional enrichment analysis and correlation of LAMRs in the TCGA-LIHC

**Figure 2:**
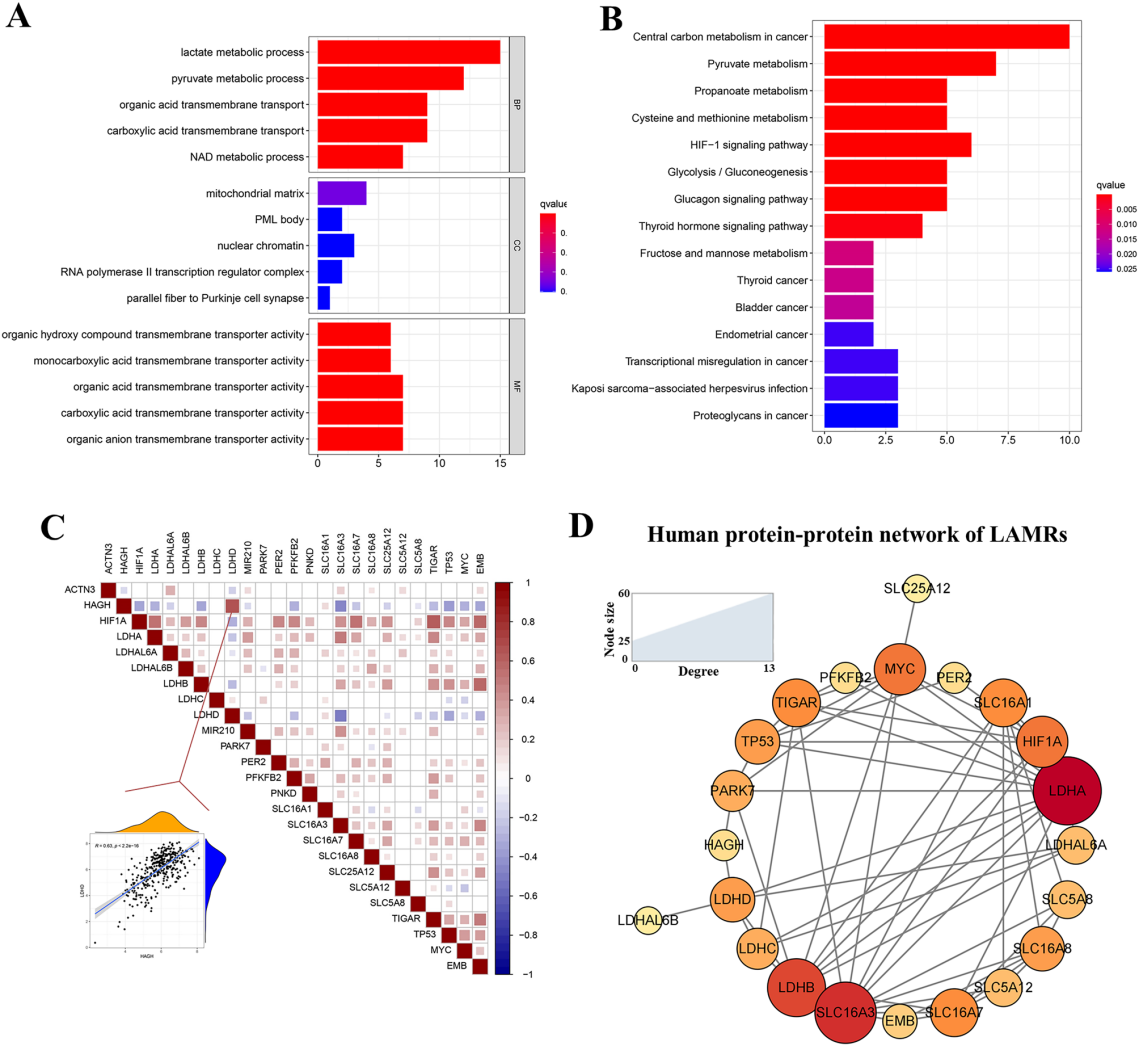
Functional enrichment analysis and correlation of LAMRs. (**A**) The top significant GO enrichment pathways. (**B**) The top significant KEGG enrichment pathways (www.kegg.jp/kegg/kegg1.html). (**C**) The correlation of LAMRs in the TCGA-LIHC cohort. (**D**) Protein–protein interaction network of LAMRs proteins.

The biological functions of LAMRs were studied by GO and KEGG analyses. The result of GO annotation indicated LAMRs were mainly enriched in lactate metabolic process, mitochondrial matrix, and organic hydroxy compound transmembrane transporter activity, which correspond to biological process (BP), cellular component (CC), and molecular function (MF), respectively (Fig. 2A). Furthermore, these LAMRs were enriched in central carbon metabolism in cancer, pyruvate metabolism, and propanoate metabolism, as suggested by KEGG enrichment analysis (Fig. 2B) (Table S3).

The relationship between LAMRs was further supported by the correlation analysis. Some correlated LAMRs regulator pairs were identified (*p* < 0.05), for example, TIGAR and HIF1A, SLC16A7 and HIF1A, and EMB and HIF1A were positive correlations. SLC16A3 and HAGH, SLC16A3, and LAHD were shown a significantly negative correlation (Fig. 2C). Taking LDHD and HAGH for example, the correlation coefficient *R* = 0.63, and *p* < 2.2e−16, which means they have a strong connection (Fig. 2C). Protein–protein network of LAMRs was shown using the STRING website and cytoscape software (Fig. 2D). The specific information for each LAMRs degree is in Table S4. LDHD, LDHB, and SLC16A3 were shown a high degree of connectivity (Fig. 2D).

### Identification of LIHC clusters by consensus clustering

**Figure 3:**
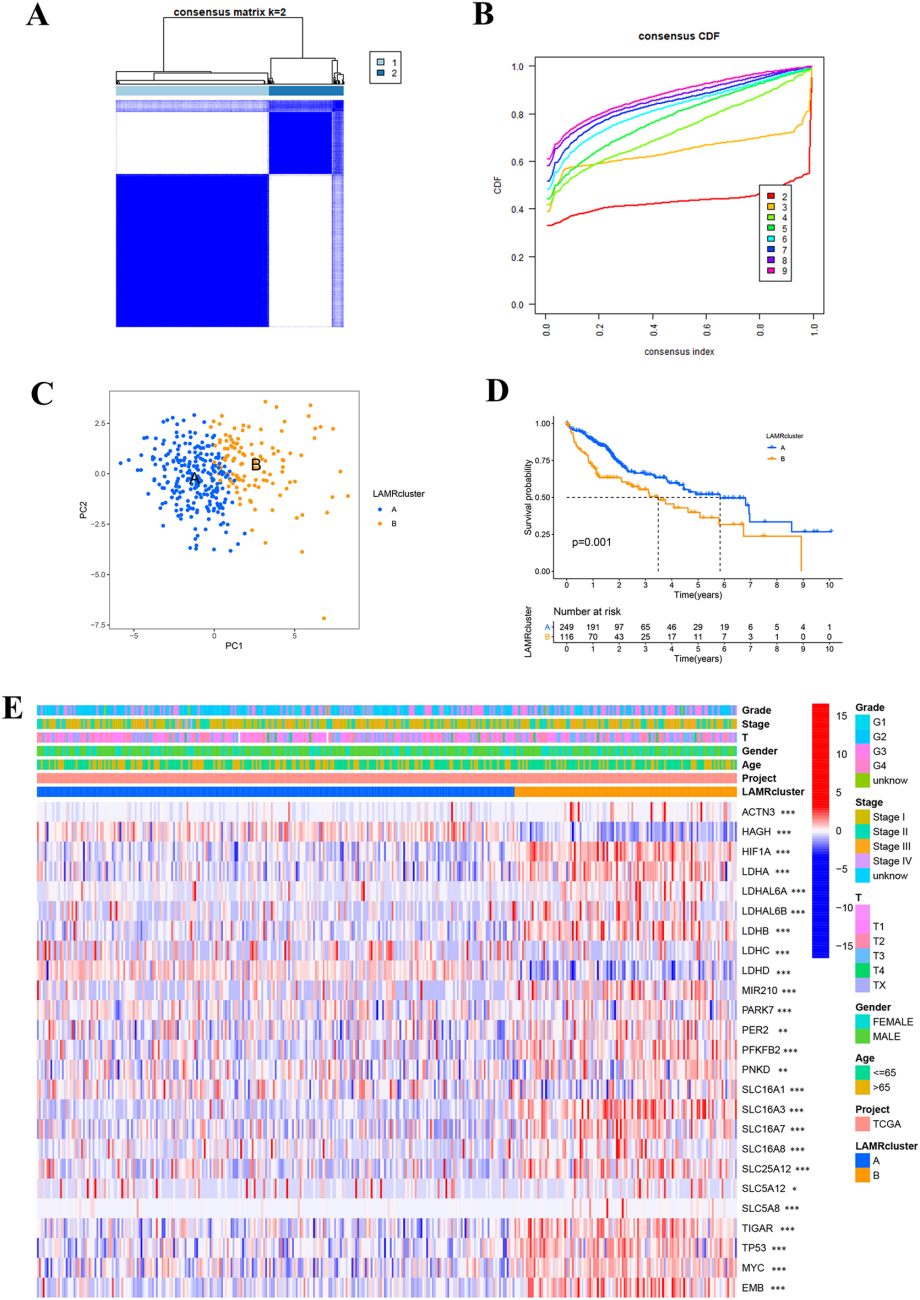
Identification of LIHC clusters using consensus clustering. (**A**) Consensus clustering matrix at K = 2. (**B**) The CDF curves for clusters at k = 2 to 9. (**C**) PCA plot for the two clusters. (**D**) Survival analysis for LIHC samples is stratified to the two clusters. (**E**) Heatmap expression of LAMRs and the clinical parameters of the two clusters.

To further understand the overall role of LAMRs in LIHC, based on the expression profiles of the 25 LAMRs, we performed consensus clustering on the 365 samples of LIHC. These patients were divided into different clusters (K = 2–9), K = 2 was taken as the optimal cluster number according to the consensus matrix (Fig. 3A), and consensus CDF curve (Fig. 3B). In addition, divided the samples into two distribution patterns using PCA (Fig. 3C). The Kaplan–Meier curves revealed that the patients in cluster A survived longer than those from cluster B (Fig. 3D). Furthermore, a heatmap was shown the differences in clinical features of the different clusters of LIHC patients and the expression of LAMRs (Fig. 3E). In cluster B, the expression of LAMRs was higher than in cluster A, e.g. HIF1A, LDHA, LDHB, SLC16A3, MYC.

### Differences in immunity between two clusters

**Figure 4:**
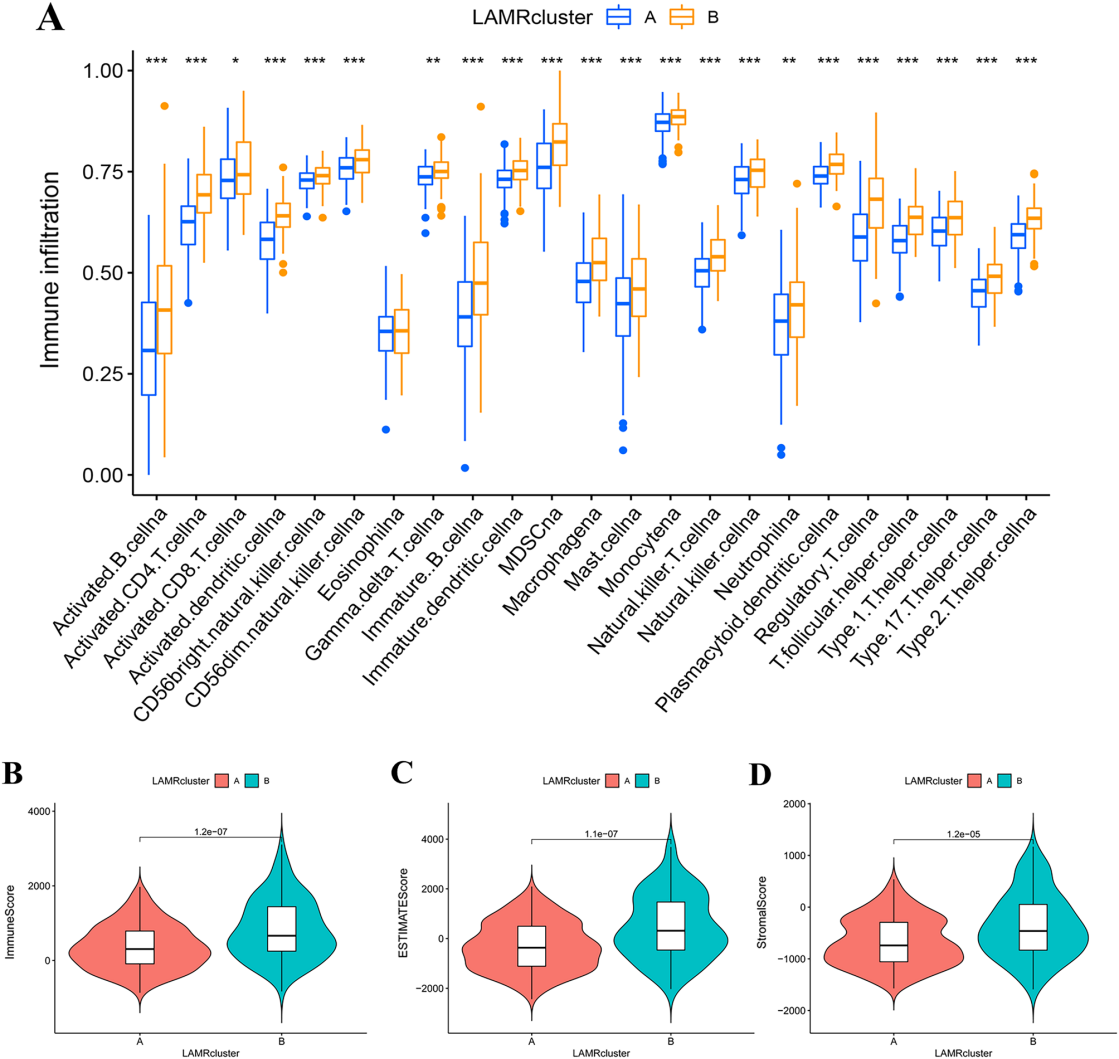
Differences in immunity between two clusters. (**A**) Abundance of 23 infiltrating immune cell types in the two HCC clusters. (**B**) Correlation between the two clusters and immune-score. (**C**) Correlation between the two clusters and ESTIMATE-score. (**D**) Correlation between the two clusters and stromal-score.

HCC immunity TME is an important factor affecting cancer progression. Therefore, the ssGSEA was used to analyze the differences in 23 kinds of immune cell infiltrations. Cluster B showed higher infiltrations of various immune cells, including the B cells, CD4+ T cells, CD8+ T cells, NK cells, and Treg cells (Fig. 4A). Besides, cluster B showed higher TME scores in Immune-score, ESTIMATE score, and Stromal-score (Fig. 4B–D).

### Identification of gene subtype

**Figure 5:**
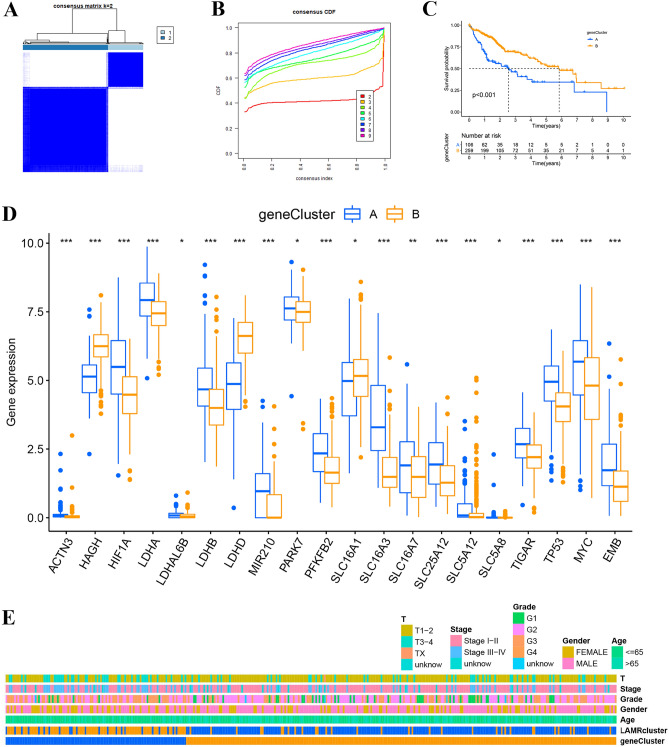
Identification of gene subtypes based on DEGs. (**A**) Consensus clustering matrix at K = 2. (**B**) The CDF curves for clusters at k = 2 to 9. (**C**) PCA plot for the two clusters. (**D**) Survival analysis for LIHC samples is stratified to the two clusters. (**E**) Comparison of 23 types of immune cells between two clusters. (**F**) Heatmap and the clinical parameters of the two clusters. *P < 0.05.

Based on the cut-off criterion we set (|LogFC|> 1.5, *P* < 0.05), a total of 335 DEGs were screened between two clusters using “limma” differential analysis (Fig. S2) **(**Table S5**)**. GO and KEGG analyses further confirmed which biological behaviors these genes were enriched in (Fig. S3) **(**Table S6**)**. Then univariate Cox regression analysis was performed to determine genes with predictive prognostic power among these 335 genes and found 194 genes associated with OS time (*P* < 0.05). Genotyping will help to optimize the treatment plan for each tumor patient and achieve precision treatment^21^. Those 194 genes were identified for unsupervised clustering analysis. LIHC patients were subsequently divided into two LAMRs gene-related clusters based on the expression of those genes (Fig. 5A, B). Cluster B was linked to a better prognosis p < 0.001 (Fig. 5C). The two LAMR gene subtypes showed significant differences in LAMRs expression (Fig. 5D). In addition, a heatmap showed the relationship between the LAMR gene-related cluster and clinical characteristics (Fig. 5E).

### Construction and validation of the prognostic LAMR risk-score

**Figure 6:**
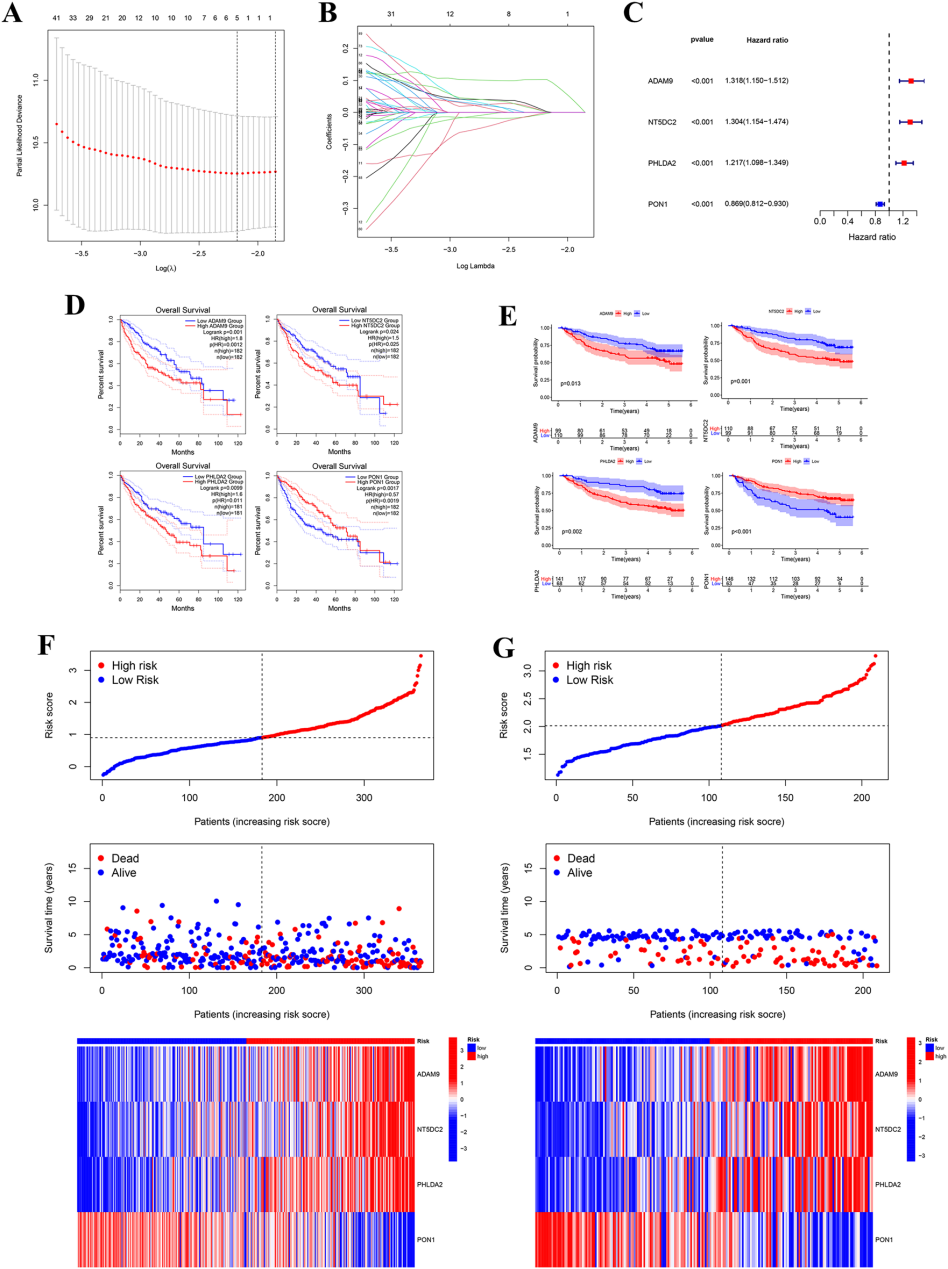
Construction and validation of the prognostic LAMR risk-score. (**A,B**). LASSO Cox regression analysis of 194 prognosis-related differentially expressed genes. (**C**) Forest plot of the four target genes that compose the risk-signature. (**D**) The Kaplan Meier analysis of the four target genes in TCGA-LIHC. (**E**) The Kaplan Meier analysis of the four target genes in GEO14520. (**F,G**) The risk score distribution, survival status, and heatmap for the expressions of the four genes in TCGA-LIHC cohort and GSE14520 cohort.

LASSO and multivariate Cox analyses were performed on 194 DEGs with subtype-associated prognosis of LAMRs to further select the optimum prognostic risk related factors (Table S7). Subsequent LASSO regression analysis was carried out, and five genes remained according to the minimum partial likelihood deviance (Fig. 6A, B). Then multivariate Cox regression analysis was performed to finally obtain four genes (ADAM9, NT5DC2, PHLDA2, and PON1). The forest map showed that PON1 was the protective factor (HR < 1), but ADAM9, NT5DC2, and PHLDA2 were risk factors (HR > 1) (Fig. 6C). We also analyzed the expression differences of the four included genes. Their expressions were obtained from the GEPIA database and found also markedly high levels of PHLDA2 and NT5DC2 in LIHC compared with normal samples (Fig. S4). We moreover examined the expression of four genes using qRT-PCR on clinical specimens (Fig. S4). The expression of NT5DC2 and PHLDA2 were consistent with the GEPIA database. PHLDA2 expression was as a representative verified using IHC and WB analysis (Fig. 11). It showed that PHLDA2 was highly expressed in human HCC tissues compared with adjacent tumor tissues. The Kaplan Meier survival analysis confirmed that high expression of ADAM9, NT5DC2, and PHLDA2 was associated with poor prognosis in patients with LIHC, however, high level of PON1 was significantly associated with better survival in patients (Fig. 6D). The same conclusion was also confirmed in GSE14520 data (Fig. 6E). For both TCGA-LIHC and GSE14520 cohort, the risk score was calculated using the following formula:

$${\text{Risk Score }} = \, \left( {0.{17}0{9 }^\ast {\text{ ADAM9}}_{{{\text{expression}}}} } \right) \, + \, \left( {0.{1641 }^\ast {\text{ NT5DC2}}_{{{\text{expression}}}} } \right) \, + \, \left( {0.{1634 }^\ast {\text{ PHLDA2}}_{{{\text{expression}}}} } \right) \, + \, \left( { - 0.0{978 }^\ast {\text{ PON1}}_{{{\text{expression}}}} } \right).$$

We also showed the distribution of risk scores and survival status among patients in TCGA-LIHC and GSE14520 (Fig. 6F, G). The mortality rate was higher in the high-risk group compared with the low-risk group. In addition, ADAM9, NT5DC2, and PHLDA2 were significantly upregulated in the high-risk group, while PON1 was significantly downregulated (Fig. 6F) (Table S8), which was consistent with the calculation of our risk score. The results of GSE14520 cohort were consistent with the above results (Fig. 6G) (Table S9).

### Association of risk score with clinical characteristics

**Figure 7:**
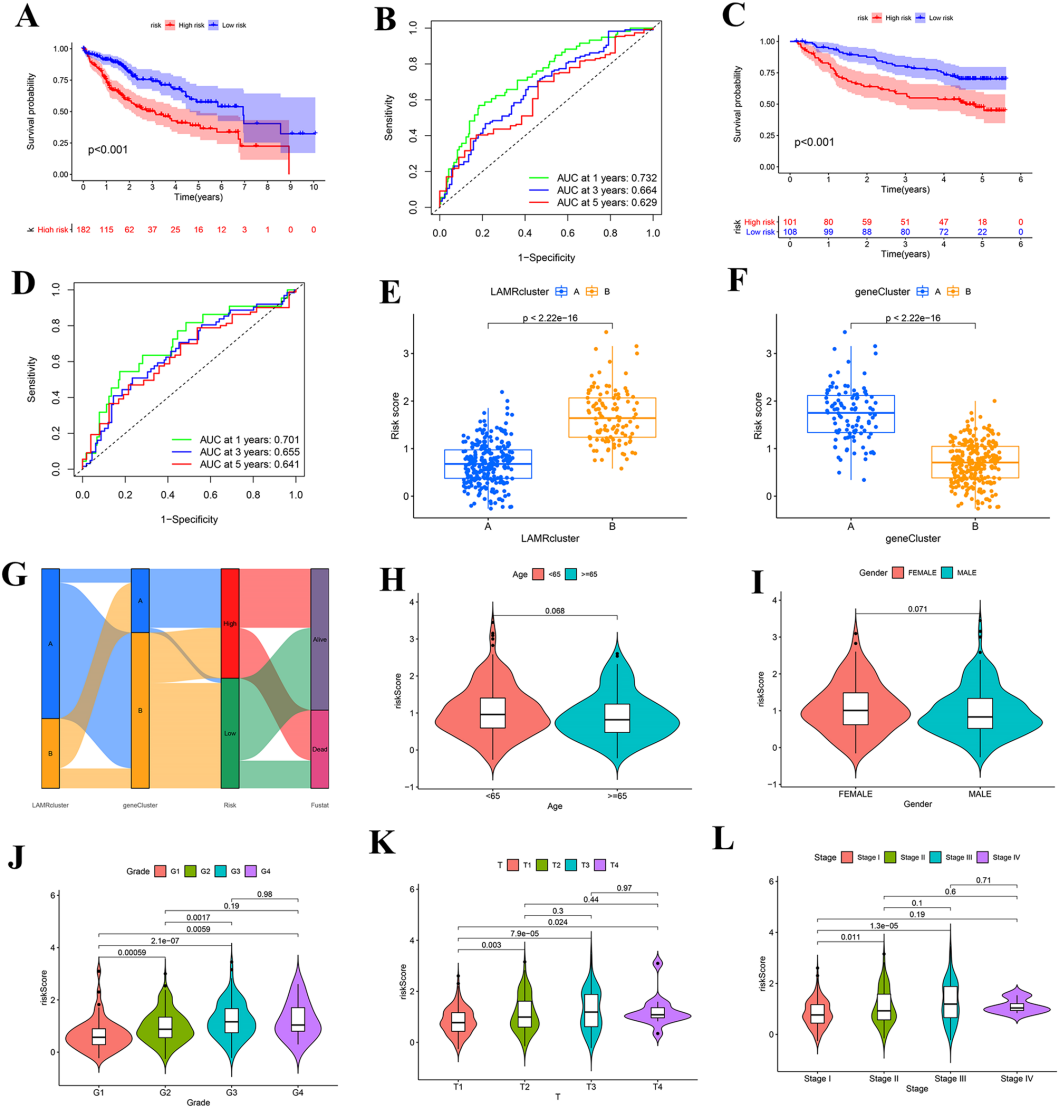
Relationship between the risk score and clinical features. (**A**) The survival analysis of risk score in TCGA-LIHC cohort. (**B**) ROC curves to predict the sensitivity and specificity of 1-, 3-, and 5-year survival according to the risk-score in TCGA-LIHC cohort. (**C**) The survival analysis of risk score in GSE14520 cohort. (**D**) ROC curves according to the risk-score in GSE14520. E. Differences in risk-score between LAMR clusters. (**F**) Differences in risk-score between gene clusters. (**G**) Mulberry figure of subtype distributions in groups with different risk-scores and survival outcomes. (**H–L**) The correlation of the risk-score with the clinical features (age, gender, grade, T stage, and stage).

The Kaplan–Meier survival analysis showed that compared to the high-risk group, the low-risk group had a better prognosis in the TCGA-LIHC group (Fig. 7A) and the GSE14520 group (Fig. 7C). Besides, the risk score also exhibits high accuracy in predicting the prognosis of HCC patients. The AUC of the risk-score was 0.732 at 1 year, 0.664 at 3 years, and 0.629 at 5 years, respectively (Fig. 7B). The AUC of the risk-score in GSE14520 was 0.701 at 1 year, 0.655 at 3 years, and 0.641 at 5 years (Fig. 7D).

We then compared the differences in risk scores between clusters, and as expected, the risk-score of cluster A was lower than cluster B (Fig. 7E). The risk score of gene subtype A was higher than subtype B, as shown in Fig. 7F. In addition, we illustrated the distribution of samples in the two LAMR clusters, two gene subtypes, risk-score groups and survival condition (Fig. 7G). Furthermore, we also analyzed the correlation of the risk-score with the clinical features in TCGA-LIHC (age, gender, grade, T stage, and stage) (Fig. 7H-L) **(**Table S10**)**. We found that the risk-score was significantly different for the grade and stage, especially G1 risk-score was significantly lower than that of high-grade patients (Fig. 7J). T stage and stage had the same result (Fig. 7K–L).

### The independent predictive ability of the risk-score and establishment of a prognostic nomogram

**Figure 8:**
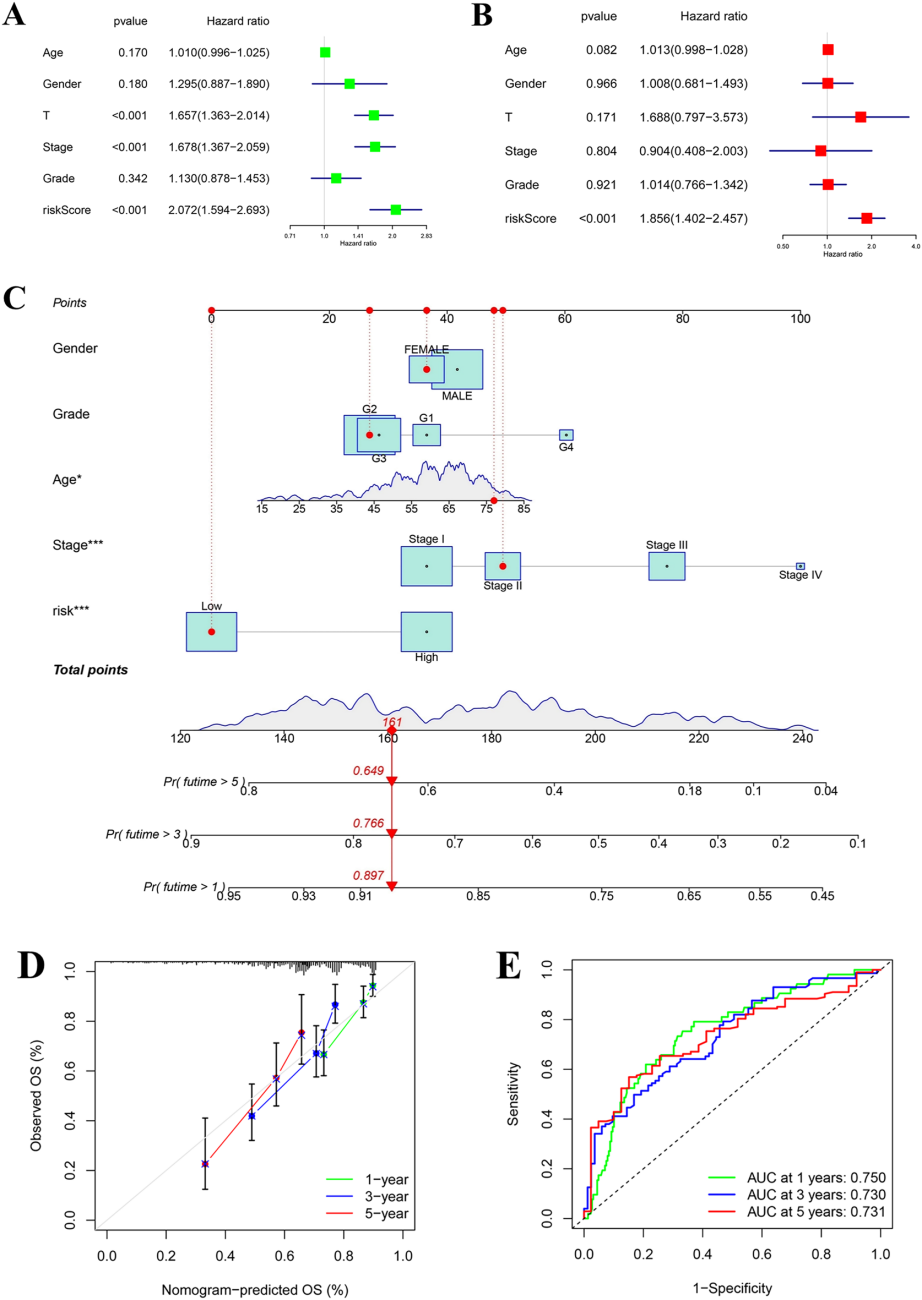
The independent predictive ability of the model and establishment of a prognostic nomogram. (**A**) Univariate Cox analysis of age, gender, T stage, stage, grade, and risk-score. (**B**) Multivariate Cox analysis of age, gender, T stage, stage, grade, and risk-score. (**C**) Establishment of a nomogram using grade, gender, age, stage, and risk. (**D**) Calibration curves of nomogram. (**E**) AUCs of nomogram at 1, 3, and 5 years.

To assess the ability of risk scores to independently predict prognosis, univariate and multivariate Cox regression analyses were carried out. T stage and stage were related to OS in univariate Cox regression analyses in the TCGA-LIHC cohort (HR = 1.657, 95% CI = 1.363–2.014, *P* < 0.001; HR = 1.678, 95% CI = 1.367–2.059, P < 0.001) (Fig. 8A). The risk-score was also significantly associated with OS (HR = 2.072, 95% CI = 1.594–2.693, *P* < 0.001) (Fig. 8A). Moreover, multivariate Cox analysis showed that the risk-score remained an independent factor (HR = 1.856, 95% CI = 1.402–2.457, *P* < 0.001) (Fig. 8B**)**.

We established a novel prognostic nomogram using the risk-score and clinical features, such as age, grade, gender, and stage (Fig. 8C). The nomogram could predict the overall survival of HCC patients. Calibration curves were plotted to visually illustrate the performance of the nomogram, showing that the predicted results were consistent with reality (Fig. 8D). ROC curve analyses showed the AUCs of 0.750 at 1 year, 0.730 at 3 years, and 0.731 at 5 years (Fig. 8E). It clearly demonstrated that the nomogram could predict the prognosis of HCC samples from the TCGA-LIHC.

### Mutation and drug susceptibility analysis

**Figure 9:**
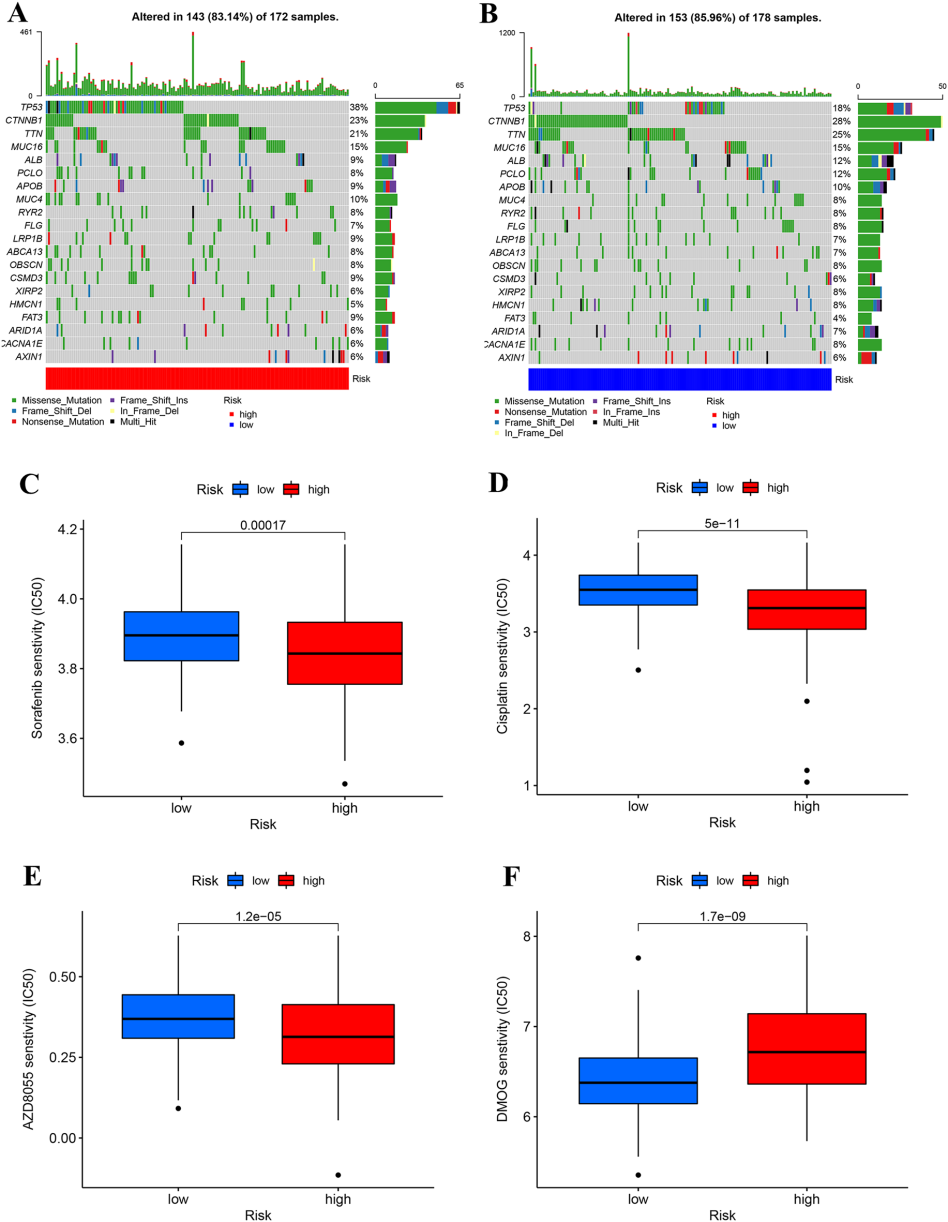
Mutation and drug sensitivity analysis. (**A**) The mutation of top 20 genes in high-risk group. (**B**) The mutation of top 20 genes in low-risk group. (**C–F**) Relationships between risk-score and drug sensitivity (**C**) sorafenib, (**D**) cisplatin, (**E**) AZD8055, (**F**) DMOG.

We also assessed differences in the top 20 gene mutations between the high- and low-risk groups. Patients in the high risk-score group had significantly higher frequencies of TP53, and FAT3 mutations compared to those in the low risk-score group. However, the mutation levels of CTNNB1 and TTN were completely opposite to those in the former (Fig. 9A, B). Next, we selected chemotherapeutic agents currently used in the treatment of LIHC, as well as future agents that may target the role of lactate regulation in the treatment of hepatocellular carcinoma, to assess the sensitivity of these agents in two group of patients. We discovered that the IC50 value of sorafenib, cisplatin, and AZD8055 was lower in the patients with the high risk-score (Fig. 9C–E), while IC50 values of DMOG were significantly lower in those with low risk-score (Fig. 9F). Totally, these results showed that risk-scores were related to drug sensitivity.

### Differential expression of ICIs and evaluation of immunotherapy response

**Figure 10:**
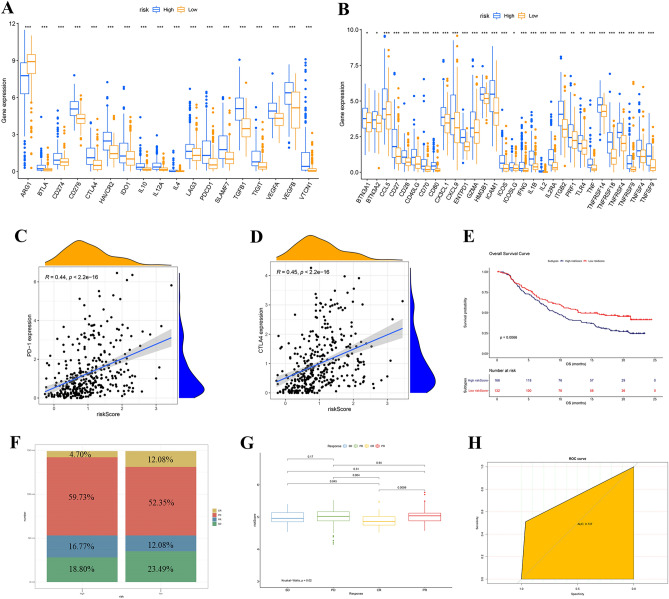
Differential expression of ICIs and evaluation of immunotherapy response. (**A,B**) The relationships between risk-score and expression of 48 ICIs. (**C**) The correlation between risk-score and PD-1 expression. (**D**) The correlation between risk-score and CTLA4 expression. (**E**) Prognostic analysis of risk-score in IMvigor210 cohort. (**F**) Rate of clinical response (CR, PR, SD, PD) to anti-PD-L1 immunotherapy in high or low risk-score groups in IMvigor210. (**G**) Differences in scores of different clinical responses. (**H**) ROC analysis of risk-score in IMvigor210.

Recently, important advances have been made in the response to ICI tumor immunotherapy of several tumors, including HCC. To verify whether risk scores correlate with immunotherapy response, we examined the expression of 48 ICIs in the high- and low-risk groups. The results showed that all ICIs were differentially expressed. Except ARG1, ICI expression in high-risk group was higher than that in low-risk group (Fig. 10A, B). PD-1 and CTLA4 were extensively studied ICIs and have been applied in clinical practice. The expression levels of above two genes were positively correlated with the risk-score (PD-1: *R* = 0.44, *P* < 2.2e−16, CTLA4: *R* = 0.45, *P* < 2.2e−16) (Fig. 10C, D). To further validate the response of this risk score to immunotherapy, we used the IMvigor210 dataset derived from immunotherapy for renal cell carcinoma. In this data set, the low-risk score had a better prognosis, which was consistent with the previous results (Fig. 10E). Rates of clinical response (CR, PR, SD, PD) to anti–PD-L1 immunotherapy in high or low risk-score groups in the IMvigor210 cohort were shown in Fig. 10F. Differences in scores of different clinical responses showed that risk-score of CR was significantly lower other three clinical responses (Fig. 10G). The ROC analysis of the IMvigor210 cohort also demonstrated that the risk-score was a predictive biomarker to immunotherapeutic benefits (AUC = 0.737, Fig. 10H).

### Validation of PHLDA2 expression in HCC

**Figure 11:**
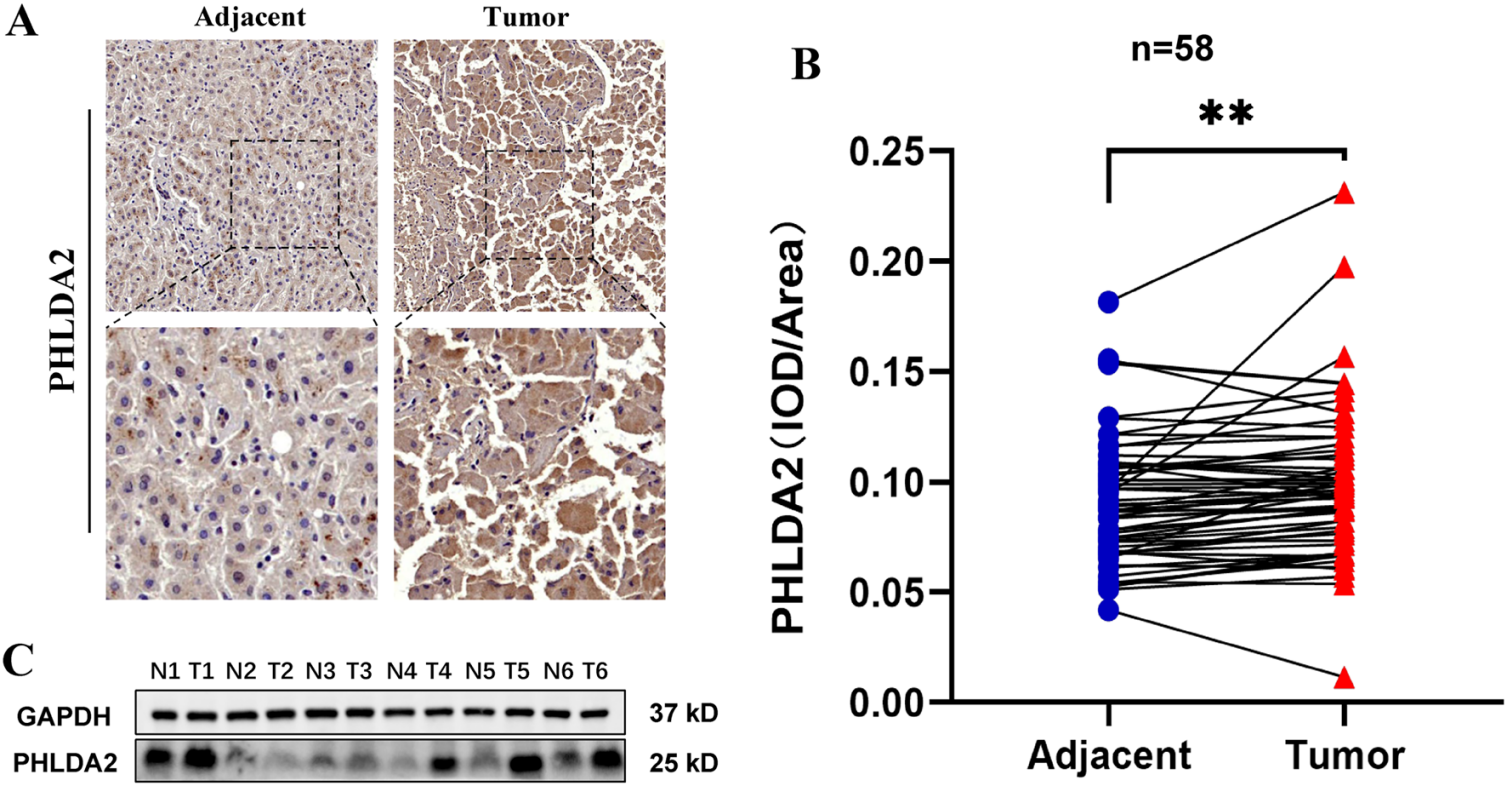
Validation of PHLDA2 expression in HCC. (**A**) Expression levels of PHLDA2 in HCC tissues and adjacent tissues by IHC. (**B**) Immunohistochemical analysis of PHLDA2 expression in 58 pairs of HCC tissues and adjacent tissues. (**C**) Western Blot analysis of PHLDA2 expression in 6 pairs of HCC tissues and adjacent tissues.

Among the four genes included in the model, PHLDA2 was selected for further verification. In our 58 patients with HCC and normal samples, PHLDA2 was significantly overexpressed in HCC (Fig. 11A, B) (Table S11). Western blot results further confirmed this result (Fig. 11C).

## Discussion

In this study, we first distinguished the expression of 25 LAMRs, a gene list containing lactate from Gene Ontology and HP, between tumor and normal tissues. The results of GO and KEGG enrichment analysis revealed that they were mainly enriched in the lactate metabolic process and central carbon metabolism in cancer, respectively. The Warburg effect is a key event in hepatocarcinogenesis, where pyruvate tends to be converted to lactate catalyzed by lactate dehydrogenase (LDH) even under aerobic conditions. LDHA is highly expressed in a variety of tumors and associated with worse overall survival^22^. MYC, and HIF-1α-mediated signaling enhances glycolysis in HCC by promoting upregulation of LDHA^23,24^. In contrast, knockdown of LDHA significantly inhibited tumor growth and metastasis of hepatocellular carcinoma^25^. Lactate is mainly transported by the monocarboxylate transporter (MCT) on the cell membrane, MCT1 (SLC16A1) mainly imports lactate, and MCT4 (SLC16A3) mainly exports lactate. MCT4 is highly expressed in HCC and promotes tumor progression, which is consistent with our results^26^. MCT1 inhibits lactate export and glucose metabolism, and inhibits HCC proliferation^27^. The strong association between these LAMRs and the progression of HCC provides a robust basis for their utilization. Further analysis suggests that LAMRs also interact with each other. There is a significant positive correlation between LDHD and HAGH expression, which suggests that both of them are involved in lactic acid metabolism. LDHD was negatively correlated with SLC16A3. In addition, LDHA, as an important protein in lactate transformation and SLC16A3 as an important protein in lactate transport, also plays a central role in LAMRs, connecting other LAMRs in tandem and jointly regulating lactate metabolism in HCC^23,28,29^. LAMRs exhibit certain genetic alterations in HCC, and TP53 has a high mutation rate, which is consistent with previous studies^30^. Copy-number changes of chromosomal segments are a common feature of human cancers and have been proposed as a driving force of tumorigenesis^31^. We found that MYC showed copy number amplification, which is consist with previous study, and MYC amplification predicts unfavorable prognosis in HCC^32,33^. However, these genetic alterations on lactate metabolism in HCC remains to be further investigated.

Thus, according to the expression of 25 LAMRs in TCGA-LIHC, we identified two distinct molecular clusters. Compared to cluster A, patients in cluster B had more advanced clinical staging, grading, and worse OS. Heatmap analysis showed that the expression of most LAMRs in cluster B was higher than that in cluster A. There were also significant differences in TME characteristics between the two clusters. The content of immune cells in cluster B including CD4 T cells, and CD8 T cells, was significantly higher than that in cluster A. Furthermore, immuneScore, ESTIMATEScore, and stromalScore also showed the same trend. Numerous studies have shown that lactate plays an integral role in tumor progression, providing a survival advantage to tumor cells through the upregulation of oncogenes, induction of angiogenesis, local infiltration of tumor cells, and distant metastasis^7,8^. Lactic acid and the resulting acidic tumor microenvironment (TME) also promote cancer cell immune evasion^7,10^. Thus, for cluster B, the inhibition of lactic acid metabolism may serve as a promising therapeutic strategy.

To further study the relationship between LAMRs and HCC patients, we identified two gene subtypes based on the DEGs between the two LAMR clusters. Next, we constructed a four-genes (ADAM9, NT5DC2, PHLDA2, and PON1) prognostic risk-score model using Cox regression and Lasso Cox regression analysis in DEGs between two LAMR cluster. It confirmed that high expression of ADAM9, NT5DC2, and PHLDA2 was associated with poor prognosis in patients with LIHC, however, high level of PON1 was significantly associated with better survival in patients. We divided the samples into high- and low-risk groups according to the median risk score. Patients in the high-risk group had a poorer prognosis, and the same result was confirmed in GSE14520. We also observed determined risk factors for tumor progression in the high-risk group of LIHC patients, including higher advanced T stage and grade. The risk score was an independent predictor of prognosis for LIHC patients, meaning that it can play a better role in predicting patient outcomes. Finally, by integrating age, gender, grade, the risk-score, and tumor stage, we established a nomogram that can moreover improve the prediction and facilitate the use of risk scores. To our satisfaction, the prognostic nomogram model can be used in the LIHC cohort and demonstrated excellent predictive ability. Four-genes expression including ADAM9, NT5DC2, PHLDA2, and PON1 were all validated in our own HCC tissues.

All four genes are closely related to the prognosis of HCC^34–36^. PON1 can regulate glucose metabolism, pentose phosphate pathway (PPP)、fatty acid oxidation (FAO)、peroxisome proliferator-activated receptor gamma (PPAR-γ) activity, and glucose metabolism via upregulation of glucose transporter-1 (GLUT-1)^37,38^. The potential involvement of ADAM9 in the regulation of lactate levels has been postulated. ADAM9, as a disintegrin and a metalloprotease 9, it has cleaved insulin B chain,and insulin-like receptor protein 5 (IGBP5) which can regulate lactate^39,40^. However, the link between other two genes (PHLDA2 and NT5DC2) and lactate metabolism remained unclear. Furthermore, additional research is required to investigate the specific impact of the four genes on the regulation of lactate.

In the early-stage HCC patients, surgical treatment, ablation, or liver transplantation are all effective treatment modalities that can significantly improve the survival time of patients. For patients with advanced HCC, systemic treatment is the only option to improve survival^41,42^. Pharmaceutical therapies to treat HCC have made great progress in recent years, including targeted tyrosine kinase inhibitors, immune based therapies, and combination of chemotherapy^43^. Sorafenib has been approved by FDA as the first line treatment for HCC ^44^. Our results showed that the high-risk score group had a higher sensitivity to sorafenib. Cisplatin, a commonly used chemotherapy drug, showed similar result. Lactate is an evolutionarily conserved metabolite and may have an effect on drug therapy for HCC. The secretion of lactic acid by tumor cells is closely related to hypoxia-inducible factor-1α (HIF-1α)^45^ and mechanistic target of rapamycin (mTOR)^46^. As expected, patients with high-risk score were more sensitive to mTOR inhibitor (AZD8055) and less sensitive to HIF-1α agonist (DMOG). Therefore, the difference in sensitivity of different subgroups to different drugs may provide precise therapeutic options for clinical patient treatment.

Although lenvatinib, like sorafenib, has recently been added as a first-line treatment for advanced liver cancer^47^, regorafenib, cabozantinib, ramucirumab were also included in second-line therapy, the prognosis of HCC after therapy is still poor^47,48^. This is partly due to the heterogeneity of HCC, but also due to drug resistance reasons. As immunotherapy becomes another major breakthrough treatment for advanced liver cancer, improving immune response and screening patients who respond well to the immune response is becoming more and more important. Studies have shown that lactate secretion and lactate shuttle in TME are involved in regulating immune response^7,49,50^. Elevated lactic acid promotes immune escape of tumor cells by increasing the accumulation of H+ in TME and maintaining a lower pH value. In CD8+ T cells, lactate reduced proliferation, cytolytic activity, and inflammatory mediator secretion^51–53^. Lactate can also suppress CD4+ cell motility, and promote T cell differentiation. Interestingly, Treg cells do not require lactic acid for survival, they use lactic acid to maintain suppressor function in TME, a high lactate condition^54^. Our result showed that LAMR cluster A had a low risk-score, a better prognosis, and lower infiltration of activated memory CD4+ and CD8+ T cells, revealing that they play a positive role in the development of HCC.

Immune checkpoint inhibitor therapy has been used in the treatment of many types of tumors. In fact, immunotherapeutic drugs for tumors, such as liver cancer, lung cancer, and melanoma, have shown beneficial results^55–57^. These immune checkpoint drugs typically exert their antitumor effects by inhibiting PD-1/PD-L1 and CTLA4. The low-risk group showed lower levels of immune checkpoint expression, including PD-1 and CTLA4, and the low-risk group had lower immune cell content above-mentioned. This may be closely related to better outcomes in the low-risk group. The overexpression of PD-L1 induces the development of an immunosuppressive TME in breast cancer^58^. PD-L1 is usually overexpressed on tumor cells to evade immune surveillance, which partly explained why the high-risk group had worse outcomes. However, it has also been reported that high PD-L1 expression can make tumor cells more sensitive to PD-1/PD-L1 inhibitors^59^. Therefore, the high-risk group may be more sensitive to the blockade of CTLA-4 and PD-1/PD-L1.

There was no doubt that the study also had some limitations. Firstly, Majority analysis were performed based on retrospective data in public databases. Due to insufficient funding, we were unable to conduct basic experimental and clinical verification for our LAMR risk-score using our own samples. Furthermore, the potential biological functions in HCC and the interaction between lactate and TME were only briefly studied, especially in terms of TME and immune cell infiltration, which need to be further investigated. Nevertheless, these current findings retain their research significance and warrant further investigation. For future work, our focus will be on the clinical implications of this risk score and validating the predictive ability of our model. Additionally, we will conduct relevant basic experiments to analyze how related genes regulate lactic acid metabolism in liver cancer, further enhancing our understanding of the role of lactate metabolism in HCC.

## Conclusions

In conclusion, we assessed the expression and prognostic significance of LAMRs, and divided TCGA samples into two clusters by consensus clustering based on LAMRs. Furthermore, we constructed risk scores based on differential genes, which can predict the prognosis of HCC patients. At the same time, the high and low risk groups also showed significant differences in mutation, drug sensitivity, immune cell infiltration, immune checkpoint genes, immunotherapy, and other aspects. Classification of tumor patients and individualized treatment will help to improve the prognosis of tumor patients^21^.

### Supplementary Information

Supplementary Figures.

Supplementary Table S1.

Supplementary Table S2.

Supplementary Table S3.

Supplementary Table S4.

Supplementary Table S5.

Supplementary Table S6.

Supplementary Table S7.

Supplementary Table S8.

Supplementary Table S9.

Supplementary Table S10.

Supplementary Table S11.

Supplementary Information.

## Data Availability

The datasets analyzed for this study can be found in the TCGA database (http://www.cancer.gov/tcga), including LIHC gene expression data (HTSeq-Counts and HTSeq-FPKM), GSE14520 (https://www.ncbi. nlm.nih.gov/geo) and IMvro210(http://research-pub.Gene.com/imvigor210corebiologies). The original data files and codes for all images and tables can be also downloaded from https://www.jianguoyun.com/p/DfFvnAIQpZvAChj1obsEIAA
