# Supplementary material for: Characteristics of lactate metabolism phenotype in hepatocellular carcinoma
Source: Sci Rep. 2023 Nov 11;13:19674. doi: 10.1038/s41598-023-47065-0 (PMC10640573; doi:10.1038/s41598-023-47065-0)
Supplement: Supplementary file 1 — Supplementary Figures. [file 41598_2023_47065_MOESM1_ESM.docx]

**
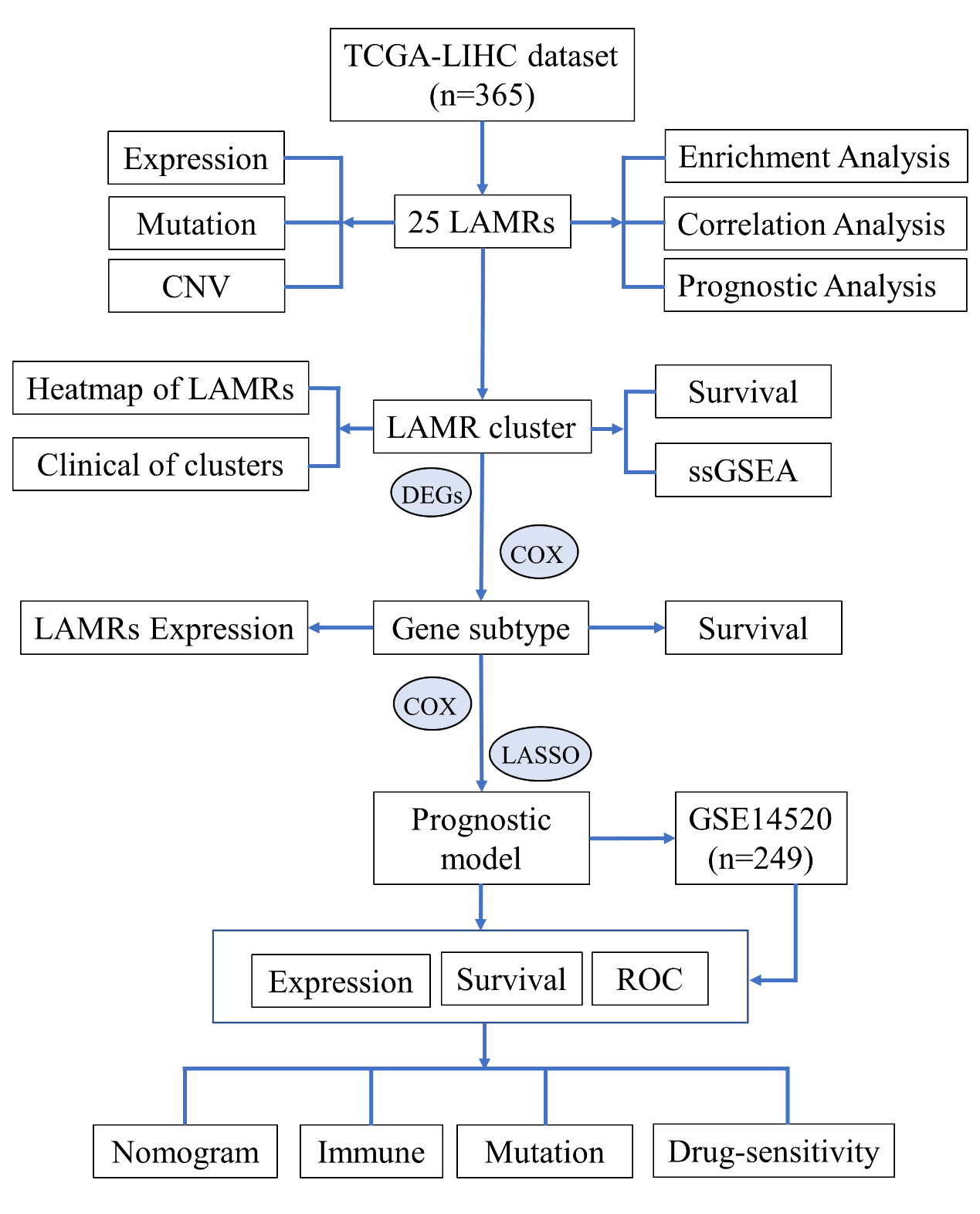
Figure S1** (A) The work flow chart of this study.

**
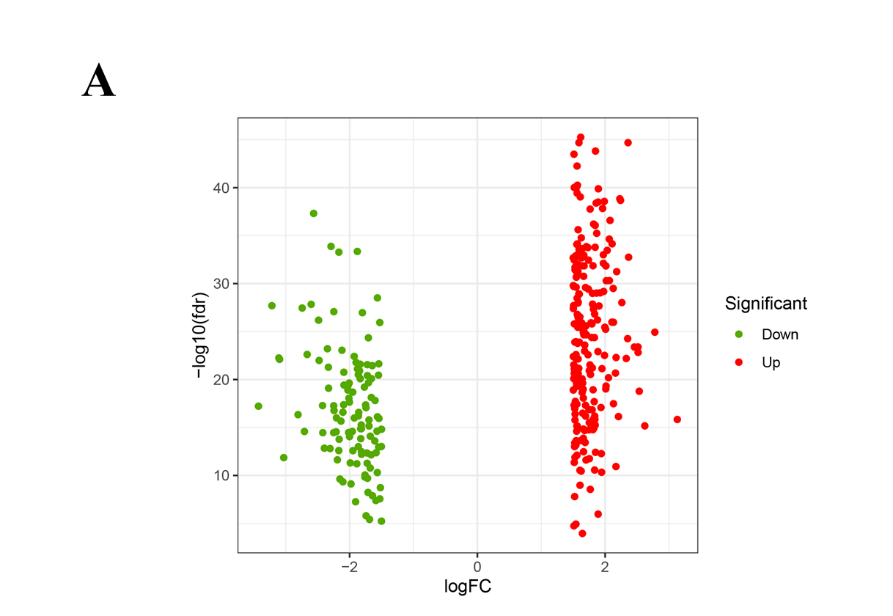
**

**Figure S2** (A) The volcano plot of 335 DEGs (|LogFC|>1.5).

**
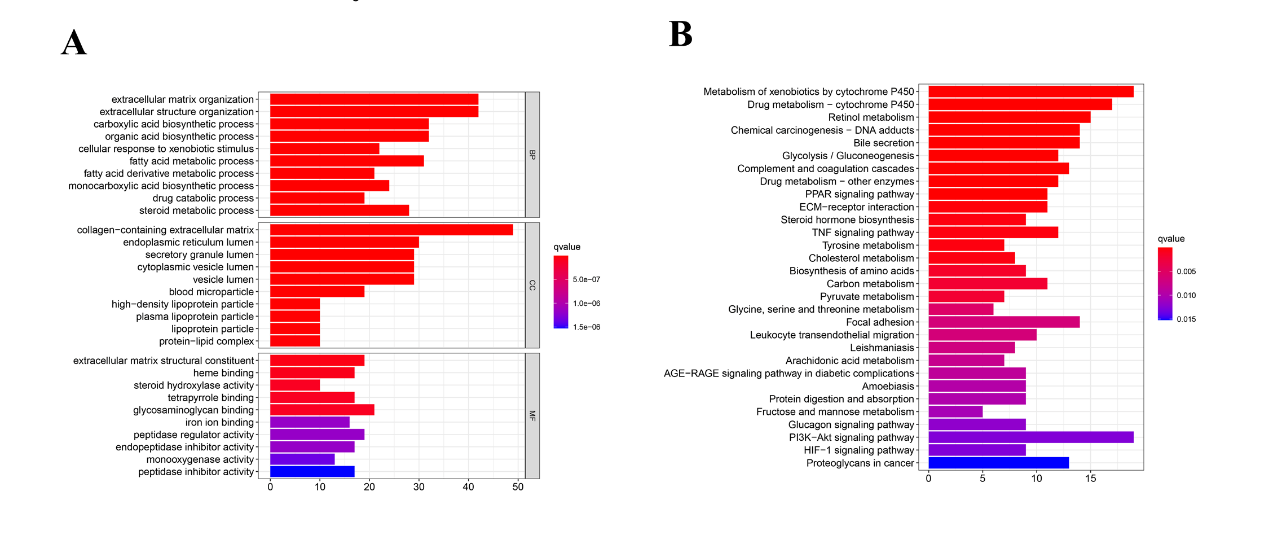
Figure S3** (A) GO [annotation](javascript:;) of 335 DEGs. (B) KEGG [annotation](javascript:;) of 335 DEGs.

**
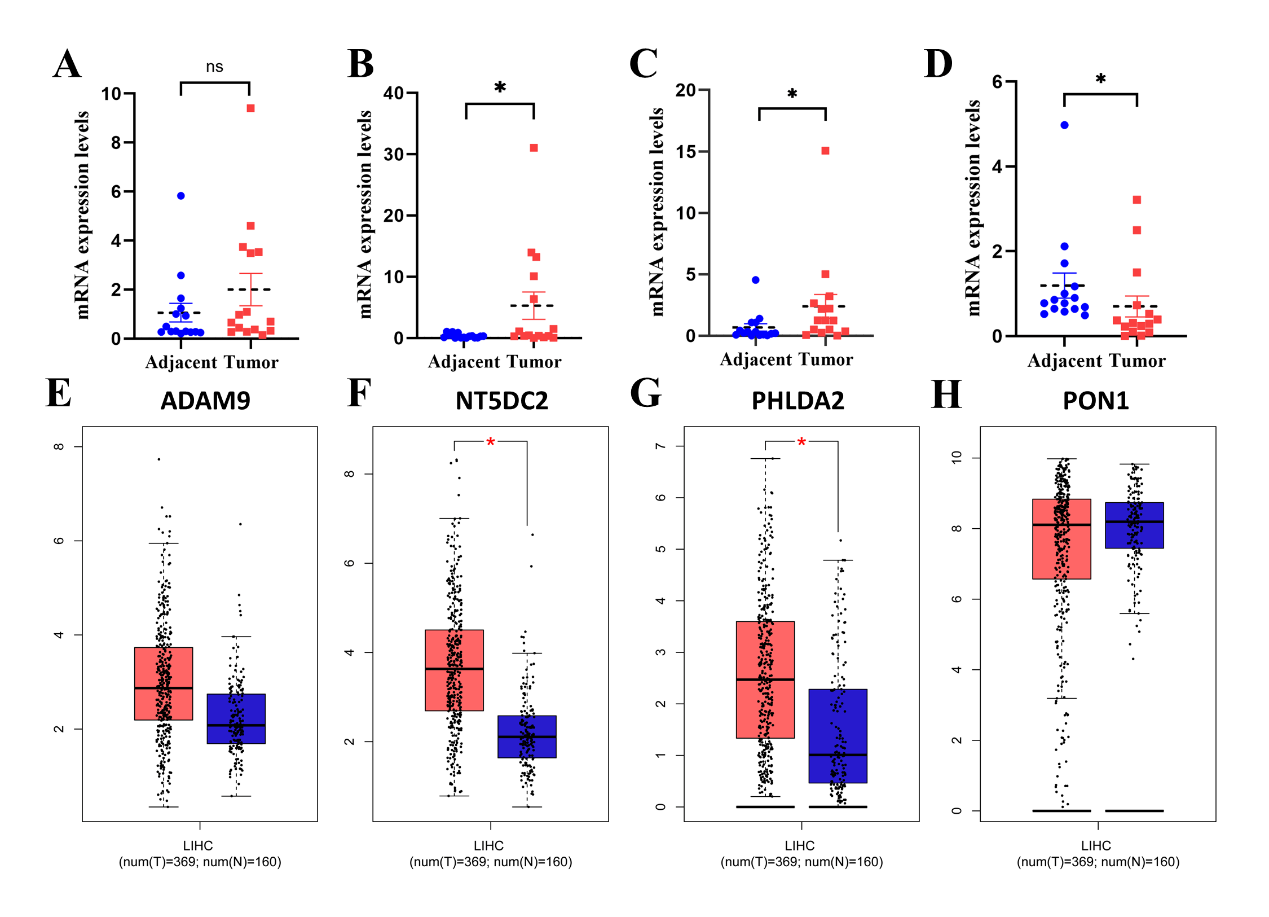
Figure S4** (A-H) Expression levels of 4 LAMR-related genes of risk score in HCC tissues and corresponding adjacent tissues by qRT-PCR (A-D) and GEPIA database (E-H).
