# Supplementary figures and images for: Characteristics of lactate metabolism phenotype in hepatocellular carcinoma
Source: Sci Rep. 2023 Nov 11;13:19674. doi: 10.1038/s41598-023-47065-0 (PMC10640573; doi:10.1038/s41598-023-47065-0)

WB supplementary(Figure 11C)


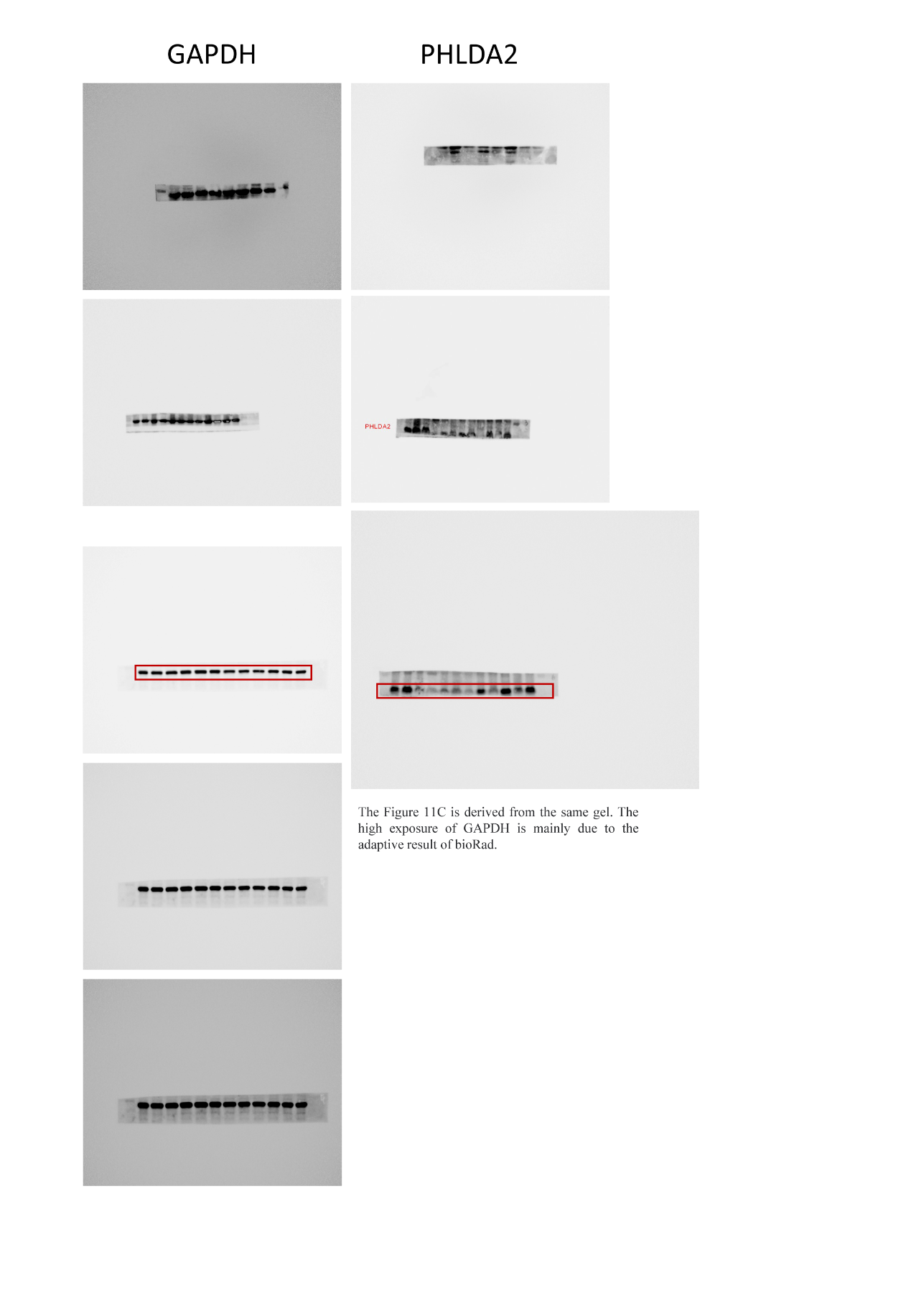

Supplement: Supplementary file 13 — Supplementary Information. [file 41598_2023_47065_MOESM13_ESM.docx]
